# The impact of infectious disease consultation in candidemia in a tertiary care hospital in Japan over 12 years

**DOI:** 10.1371/journal.pone.0215996

**Published:** 2019-04-25

**Authors:** Masahiro Ishikane, Kayoko Hayakawa, Satoshi Kutsuna, Nozomi Takeshita, Norio Ohmagari

**Affiliations:** 1 Disease Control and Prevention Center, National Center for Global Health and Medicine, Shinjuku-ku, Tokyo, Japan; 2 AMR Clinical Reference Center, National Center for Global Health and Medicine, Shinjuku-ku, Tokyo, Japan; Azienda Ospedaliera Universitaria di Perugia, ITALY

## Abstract

**Background:**

Candidemia is one of the major causes of morbidity and mortality as a hospital acquired infection. Infectious diseases consultation (IDC) might be beneficial to improve candidemia outcomes; however, only limited data from short periods of time are available thus far.

**Methods:**

An observational study of all candidemia patients at a large tertiary care hospital between 2002 and 2013 was conducted. A candidemia episode was defined as ≥ 1 positive result for *Candida* spp. in blood culture. Patients who died or transferred to another hospital within two days after their first positive blood culture were excluded. Independent risk factors for 30-day mortality were determined.

**Results:**

Among 275 patients with 283 episodes of candidemia, 194 (68.6%) were male, and the mean age was 70.0 ± 15.8 years. Central line-associated bloodstream infections, peripheral line-associated bloodstream infections, intra-abdominal infection, and unknown source comprised 220 (77.7%), 35 (12.4%), 13 (4.7%), and 15 (5.3%) episodes, respectively. A total of 126 patients (44.5%) received IDC. Factors independently associated with 30-day mortality in patients with candidemia were urinary catheters use (adjusted hazard ratio [HR] = 2.94; 95% confidence interval [CI] = 1.48–5.87; *P* = 0.002) and severe sepsis/septic shock (adjusted HR = 2.10; 95% CI = 1.20–3.65; *P* = 0.009). IDC was associated with a 46% reduction in 30-day mortality (adjusted HR = 0.54; 95% CI = 0.32–0.90; *P* = 0.017).

**Conclusion:**

IDC was independently associated with a reduction in 30-day mortality. Only 44.5% of patients with candidemia in this cohort received IDC. Routine IDC should be actively considered for patients with candidemia.

## Introduction

Candidemia is one of the major causes of morbidity and mortality as hospital acquired infection, [1–5] and high overall mortality rate was reported with 25–60% [6–8]. Additionally, candidemia is related to the extended hospitalization and increased health care costs [6–8]. Clinical intervention by infectious diseases consultation (IDC) has been shown to reduce morbidity and/or mortality in several infections [9–12], including candidemia [13–16]. Underscoring the value of IDC as antifungal stewardships is important.

However, previous studies are limited by small patient sample sizes and short time periods of patient inclusion and/or follow up [13–16]. Moreover, studies of the survival outcome effects of IDC on candidemia in Asian countries, especially in Japan, are few.

In the past study [17], we reported that peripheral-line associated candidemia (PLAC) was an important cause of candidemia in the healthcare settings. Although IDC was associated with the predictors of PLAC, we did not evaluate the survival outcome of IDC on candidemia [17]. Thereby, we conducted a retrospective cohort study covering a 12-year period to evaluate the relationship between survival outcomes of candidemia and IDC in a tertiary care hospital in Japan as advances on previous work [17].

## Materials and methods

### Hospital setting and study design

A retrospective cohort study of all candidemia was conducted between January 2002 and December 2013 at the National Centre for Global Health and Medicine (NCGM), which has approximately 780 inpatient beds and serves as a tertiary referral hospital for metropolitan Tokyo. This study was approved by the ethics committee of the NCGM (approval no: NCGM-G-001589-00) and was implemented in accordance with the Declaration of Helsinki. Patient information was anonymized and deidentified prior to analysis. Due to the retrospective nature of the study, patient consent was waived.

### Data source

We identified all cases of candidemia using the microbiological laboratory database. The parameters were collected from patient charts included the following: (i) demographics including time period variable which was divided 2002–2009 and 2010–2013 due to the establishment of the official infectious disease consultation service with five infectious disease specialists in 2010; (ii) immunosuppressive status; (iii) background and comorbid conditions; (iv) recent healthcare-associated exposures; (v) recent exposure to antibiotic and antifungal therapy; (vi) infection-related characteristics; (vii) the severity of illness (sepsis, severe sepsis, and septic shock) and haematogenous dissemination; (viii) antifungal therapy against candidemia; (ix) outcome (clinical failure, persistent candidemia, in-hospital and 30-day/180-day mortality, discharge to a long term care facility (LTCF), re-admission, length of hospital stay after candidemia (excluding those who died), and duration of candidemia, as well as past our study [17–19]. Persistent candidemia means the case with follow up-blood culture positive of *Candida*. spp., after 72hr with empirical therapy [17]. Duration of candidemia was calculated from the date when the initial blood culture positive of *Candida*. spp. was drawn, to the date when the follow-up blood culture negative of *Candida*. spp was drawn [17]. Additionally, we reviewed IDC for management of candidemia and whether or not recommended candidemia treatment including examination for endophthalmitis and endocarditis were performed.

### Definitions of variables including candidemia episode

We defined an episode of candidemia as isolation of *Candida* spp. from at least one peripherally-taken blood culture in a patient with clinical signs and symptoms of infection [17, 20]. When caused by different *Candida* spp. or occurring at least 30 days apart, with improvement of clinical features of infection and at least one negative blood culture in the period [17, 21], we considered that episodes of candidemia were separated. We excluded the episodes identified within 48 hours of hospital admission, because these episodes were thought not to be hospital acquired, and determining important candidemia parameters, such as duration, would be difficult [17]. Additionally, episodes who died or transferred to another hospital within 2 days after their first positive blood culture were also excluded, due to the limited opportunity for evaluating IDC effects.

Central line-associated bloodstream infections (CLABSI) and intra-abdominal infection (IAI) were defined according to the National Healthcare Safety Network Surveillance definition and the guidelines of the Infectious Diseases Society of America (IDSA) [17, 20, 22, 23]. We defined peripheral line-associated bloodstream infections (PLABSI) as the presence of at least one of the following conditions: (i) the presence of phlebitis, and/or (ii) resolution of clinical symptoms after short-term peripheral line withdrawal with a careful exclusion of another focus of bacteraemia [17, 24]. We defined empiric and definitive therapy as administration of systemic antifungal drugs within 72 hours of the onset of candidemia, and based on the guideline of IDSA [17, 20]. The variables related therapy (the time to antifungal therapy, adequate source control, time to central or peripheral vein catheter removal, and clinical failure) were defined as past studies [17, 25]

### Infectious disease consultation and appropriateness of an antifungal therapy or duration

IDC was recommended for patients with candidemia as per hospital policy, and was performed when requested by the primary physician in charge. Request for consultation was not mandatory. IDC comprised chart review, physical examination of the patient, a follow-up visit, and written recommendations for therapy based on published IDSA guidelines [20]. Individual case discussion was performed with the primary physician in charge. We evaluated whether antifungal therapy, including the duration, was in accordance with the published IDSA guidelines [20].

### Microbiological data

*Candida* spp. from positive blood culture were identified using API 20 C AUX (Biomerieux Japan Co., Ltd., Japan), and ID 32 C (Biomerieux Japan Co., Ltd., Japan). Antifungal susceptibility testing was performed using the commercially prepared colorimetric microdilution panel (ASTY; Kyokuto Pharmaceutical Industrial Co., Ltd.). which was developed according to the CLSI recommendation. During the study period, there were no changes to the microbiological identification and susceptibility testing process.

### Statistical analysis

Continuous variables were shown as the mean ± standard deviation (SD) or the median with interquartile range (IQR), and compared using Student’s t-test or Mann-Whitney U test. Categorical variables were shown as absolute and relative frequencies, and compared using the χ^2^ test or Fisher’s exact test.

We compared demographic characteristics, clinical factors, and outcomes between episodes with and without IDC, using logistic regression univariate analysis with odds ratios (OR) and 95% confidence intervals (CI). Multivariable survival analyses were performed and predictive models for 30-day and 31-180-day all-cause mortality were built.

For the 30-day and 31-180-day mortality models, Cox proportional hazards models were applied. We considered the potential predictive factors with a *P*-value of < 0.10 in the univariate analysis, or that were hypothesized *a priori* to be clinically or epidemiologically important, for inclusion in the multivariate model for predictive factors. The relationship of the variable of IDC and mortality during the 180-day follow up period was illustrated using a Kaplan-Meier estimator. Survival characteristics were compared between groups with and without IDC using the log-rank test. We defined statistical significance as a 2-sided *P*-value of < 0.05, and all statistical analyses were performed with SPSS Version 24 (IBM Corp., Armonk, NY, USA).

## Results

### Description of candidemia from 2002 to 2013

The 12-year study period included a total of 283 episodes of candidemia from 275 patients. The overall incidence of candidemia episodes from 2005–2013 was 0.10/1000 patient-days and 1.64/1000 hospital admissions. The mean age of this cohort was 70.0 ± 15.8 years and 194 (68.6%) were male. One hundred twenty-six patients (44.5%) received IDC. CLABSI, PLABSI, IAI, and unknown source of infection consisted of 220 (77.7%), 35 (12.4%), 13 (4.7%), and 15 (5.3%), respectively. From the 283 episodes of candidemia, 295 *Candida* spp. were collected, including 25 episodes of polymicrobial bacteraemia/fungaemia due to different species of *Candida* spp. (12 episodes) or to pathogens other than *Candida* spp. (13 episodes). *Candida* spp. were comprised of 131 (44.4%) isolates of *C*. *albicans*, 74 (25.1%) of *C*. *glabrata*, 45 (15.3%) of *C*. *parapsilosis*, 28 (9.5%) of *C*. *tropicalis*, and 17 (5.8%) of other *Candida* spp., including *C*. *krusei* (n = 4), *C*. *guilliermondii* (n = 3), *C*. *lusitaniae* (n = 2), *C*. *dubliniensis* (n = 1), and unclassified (n = 7).

### Comparison of candidemia patients with and without IDC

Comparison of candidemia patients with and without IDC is summarized in Table 1.

**Table 1:** Comparison of candidemia patients with and without infectious disease consultation, n = 283.

Category	Variable	With IDC		Without IDC		OR (95% CI)		*P* value
		(n = 126, 44.5%)		(n = 157, 55.1%)				
Demographics	Mean age (years) ± SD	69.1	± 16.4	70.7	± 15.4			0.38
	Females	35	(27.8)	54	(34.4)	0.73	(0.44–1.22)	0.23
	Candidemia from 2010 to 2013	70	(55.6)	33	(21.0)	4.70	(2.79–7.90)	<0.001
Immunocompromised status	HIV infection	9	(7.1)	1	(0.6)	12.00	(1.50–96.04)	0.006
	Neutropenia (< 0.5 × 10^9^ cells/L) at onset	2	(1.6)	5	(3.2)	0.49	(0.094–2.57)	0.47
	Chemotherapy in the past month	29	(15.9)	29	(18.5)	0.83	(0.45–1.56)	0.57
	Steroid therapy in the past month	26	(20.6)	25	(15.9)	1.37	(0.75–2.52)	0.31
	Radiation therapy in the past month	6	(4.8)	12	(7.6)	0.60	(0.22–1.66)	0.32
	Transplantation in the past month	3	(2.4)	1	(0.6)	3.81	(0.39–37.03)	0.33
Background and comorbid conditions on admission	Dependent functional status	52	(41.3)	69	(43.9)	0.90	(0.56–1.44)	0.65
	Charlson's weighted index comorbity score (6), mean ± SD	4.1	± 2.7	4.3	± 3.0			0.56
	Diabetes mellitus	36	(28.6)	40	(25.5)	1.17	(0.69–1.98)	0.56
	Solid-organ cancer within last 1 year	42	(33.3)	69	(43.9)	0.64	(0.39–1.04)	0.069
	Hematological malignancy within last 1 year	13	(10.3)	12	(7.6)	1.39	(0.61–3.16)	0.43
	Chronic kidney disease stage V	10	(7.9)	8	(5.1)	1.61	(0.61–4.20)	0.33
	Liver diseases	3	(2.4)	11	(7.0)	0.32	(0.088–1.19)	0.099
	Chronic heart disease	28	(22.0)	31	(19.7)	1.16	(0.65–2.06)	0.61
	Chronic obstructive pulmonary disease	11	(8.7)	22	(14.0)	0.59	(0.27–1.26)	0.17
	Cerebrovascular disease	31	(24.6)	32	(20.4)	1.28	(0.73–2.24)	0.40
	Dementia	6	(4.8)	14	(8.9)	0.51	(0.19–1.37)	0.18
	Connective tissue disease	12	(9.5)	4	(2.5)	4.03	(1.27–12.81)	0.018
	Peptic ulcer disease	9	(7.1)	17	(10.8)	0.63	(0.27–1.47)	0.29
	Peripheral vascular disease	2	(1.6)	2	(1.3)	1.25	(0.17–9.00)	1.00
	Hemiplegia	12	(9.5)	12	(7.6)	1.27	(0.55–2.94)	0.57
Recent health care-associated exposures before onset of candidemia	Resided LTCF in the past 3 months	8	(6.3)	6	(3.8)	1.71	(0.58–5.05)	0.33
	Hospitalized in the past 3 months	53	(42.1)	77	(49.0)	0.75	(0.47–1.21)	0.24
	Invasive procedure/surgery in the past 3 months	46	(36.5)	54	(34.4)	1.10	(0.67–1.79)	0.71
	Tracheotomy in the past 3 months	16	(12.7)	15	(9.6)	1.38	(0.65–2.91)	0.40
	Urinary catheters (for ≥ 2 days) at onset of candidemia	86	(68.3)	93	(59.2)	1.48	(0.91–2.42)	0.12
	CVC (for ≥ 2 days) at same onset	98	(77.8)	133	(84.7)	0.63	(0.35–1.16)	0.13
	Median days of CVC prior to onset of candidemia (IQR)	11	(4–24)	14	(6–23)			0.19
	Undergoing haemodialysis in the past month	13	(10.3)	9	(5.7)	1.89	(0.78–4.58)	0.15
	Transfusion in the past month	67	(53.2)	87	(55.8)	0.90	(0.56–1.44)	0.66
	TPN in the past month	75	(59.5)	97	(61.8)	0.91	(0.56–1.47)	0.70
	Median days of TPN prior to onset of candidemia (IQR)	6	(0–15)	8	(0–19)			0.35
	ICU stay in current hospitalization before onset of candidemia	24	(19.0)	12	(7.6)	2.84	(1.36–5.95)	0.004
	Median hospital days prior to the onset of candidemia (IQR)	40	(20–67)	31	(18–62)			0.20
Exposure to antibiotic therapy (for ≥ 3 days) prior to isolation of *Candida* spp.	Over all	120	(95.2)	147	(93.6)	1.36	(0.48–3.85)	0.56
	Penicillins^a^	61	(48.4)	61	(38.9)	1.48	(0.92–2.37)	0.11
	Cephalosporins^b^	59	(46.8)	71	(45.2)	1.07	(0.67–1.71)	0.79
	Carbapenems	73	(57.9)	68	(43.3)	1.80	(1.12–2.90)	0.014
	Fluoroquinolones	36	(28.6)	31	(19.7)	1.63	(0.94–2.82)	0.083
	Aminoglycosides	13	(10.3)	15	(9.6)	1.09	(0.50–2.38)	0.83
	Trimethoprim-sulfamethoxazole	19	(15.1)	14	(8.9)	1.81	(0.87–3.78)	0.11
	Clindamycin	14	(11.1)	17	(10.8)	1.03	(0.49–2.18)	0.94
	Metronidazole	16	(12.7)	8	(5.1)	2.71	(1.12–6.56)	0.023
	Glycopeptide	46	(36.5)	41	(26.1)	1.63	(0.98–2.70)	0.060
Exposure to antifungal therapy (for ≥ 3 days) prior to isolation of *Candida* spp.	Overall	20	(15.9)	18	(11.5)	1.46	(0.73–2.89)	0.28
	Fluconazole	4	(3.2)	8	(5.1)	0.61	(0.18–2.08)	0.56
	Micafungin	15	(11.9)	7	(4.5)	2.90	(1.14–7.34)	0.020
	Voriconazole	2	(1.6)	0	(0.0)			0.20
	Liposomal amphotericin b	4	(3.2)	0	(0.0)			0.038
	Itraconazole	3	(2.4)	5	(3.2)	0.74	(0.17–3.16)	0.74
Microbiology	*Candida* species:							
	*C*. *albicans*	62	(49.2)	69	(43.9)	1.24	(0.77–1.98)	0.38
	*C*. *glabrata*	29	(23.0)	45	(28.7)	0.74	(0.43–1.28)	0.28
	*C*. *parapsilosis*	21	(16.7)	24	(15.3)	1.11	(0.59–2.10)	0.75
	*C*. *tropicalis*	13	(10.3)	15	(9.6)	1.09	(0.50–2.38)	0.83
	Others^c^	7	(5.6)	10	(6.4)	0.87	(0.32–2.34)	0.78
	Polymicrobial bacteremia/fungemia^d^	17	(12.5)	8	(5.1)	2.91	(1.21–6.97)	0.013
	Previous *Candida* colonization within a week before candidemia	45	(35.7)	45	(28.7)	1.38	(0.84–2.29)	0.21
Source of infection	CLABSI	93	(73.8)	127	(80.9)	0.67	(0.38–1.17)	0.16
	PLABSI	22	(17.5)	13	(8.3)	2.34	(1.13–4.87)	0.020
	Intra-abdominal infection	8	(6.3)	5	(3.2)	2.06	(0.66–6.46)	0.21
	Unknown source	3	(2.4)	12	(7.6)	0.30	(0.081–1.07)	0.062
Severity of illness indices at the time of candidemia	Sepsis	48	(38.1)	44	(28.0)	1.58	(0.96–2.61)	0.072
	Severe sepsis	46	(36.5)	62	(39.5)	0.88	(0.54–1.43)	0.61
	Septic shock	19	(15.1)	20	(12.8)	1.21	(0.61–2.38)	0.59
	Severe sepsis/septic shock	65	(51.6)	82	(55.2)	0.98	(0.61–1.56)	0.91
	Reduced consciousness	12	(9.5)	21	(13.4)	0.68	(0.32–1.45)	0.32
	Acute mechanical intubation/ventilation	17	(13.5)	14	(8.9)	1.59	(0.75–3.37)	0.22
	Developed acute renal failure	32	(25.4)	24	(15.3)	1.89	(1.04–3.41)	0.034
	Developed acute liver injury	38	(30.2)	57	(36.3)	0.76	(0.46–1.25)	0.28
	Chorioretinitis	18	(14.3)	15	(9.6)	1.58	(0.76–3.27)	0.22
Therapy	Empirical antifungal therapy within 72 hours of the onset of candidemia								
	Fluconazole	25	(19.8)	59	(37.6)	0.41	(0.24–0.71)	0.001
	Micafungin	92	(73.0)	77	(49.0)	2.81	(1.70–4.65)	<0.001
	Voriconazole	0	(0.0)	1	(0.6)			1.00
	Liposomal amphotericin b	7	(5.6)	4	(2.5)	2.25	(0.64–7.87)	0.23
	None	1	(0.8)	16	(10.2)	0.071	(0.009–0.54)	0.001
	Change of antifungal drugs due to clinical failure	18	(14.3)	14	(8.9)	1.70	(0.81–3.57)	0.16
Complications	Overall	26	(20.6)	20	(12.7)	1.78	(0.94–3.37)	0.07
	Endophthalmitis	18	(14.3)	15	(9.6)	1.58	(0.76–3.27)	0.22
	Endocarditis	1	(0.8)	0	(0.0)			0.45
	Pulmonary embolism	2	(1.6)	0	(0.0)			0.20
	Vertebral osteomyelitis	1	(0.8)	0	(0.0)			0.45
	Thrombosis	4	(3.2)	5	(3.2)	1.00	(0.26–3.79)	1.00
Outcome	Clinical failure	26	(19.8)	43	(27.4)	0.66	(0.37–1.15)	0.14
	Persistent candidemia for ≥ 72 hours of therapy	122	(96.8)	127	(80.9)	7.21	(2.47–21.06)	<0.001
	In-hospital mortality	47	(37.3)	66	(42.0)	0.82	(0.51–1.33)	0.42
	30-day mortality	23	(18.3)	44	(28.0)	0.57	(0.32–1.02)	0.055
	Early (< 72 hours)	0	(0.0)	1	(0.6)			1.00
	Non-early (days 3–30)	23	(18.3)	43	(27.4)	0.59	(0.33–1.05)	0.071
	90-day mortality	41	(32.5)	64	(40.8)	0.70	(0.43–1.14)	0.16
	180-day mortality	57	(45.2)	76	(48.6)	0.88	(0.55–1.41)	0.60
	Discharged to LTCF after being admitted from home	40	(31.7)	45	(28.7)	1.16	(0.70–1.93)	0.57
	Additional hospitalizations in 6 months after completed candidemia therapy	22	(17.5)	27	(17.2)	1.02	(0.55–1.89)	0.95
	Median total LOS days (IQR)	97	(68–148)	71	(46–110)			<0.001
	Median LOS after candidemia day (IQR)	52	(26–83)	31	(15–54)			<0.001
	Median LOS after candidemia day, excluding those who died (IQR)	31	(0–69)	18	(0–43)			0.017
	Median duration candidemia days (IQR)	8	(5–12)	5	(0–11)			<0.001

Unless otherwise stated, data are presented as n (%)

IDC, infectious disease consultation; OR, odds ratio; CI, confidence interval; SD, standard deviation; LTCF, long term care facility; CVC, central venous catheter; IQR, interquartile range; TPN total parenteral nutrition; CLABSI, central line-associated blood stream infection; PLABSI, peripheral line-associated blood stream infection; LOS, length of hospital stay

^a^Included ampicilline, sulbactam/ampicilline, piperacillin, and tazobactam/piperacillin

^b^Included ceftriaxone, ceftazidime, and cefepime.

^c^Other *Candida* species were included *C*. *gelliermondii* (with IDC, 2; without IDC, 1), *C*. *lusitaniae* (2 in with IDC), *C*. *krusei* (with IDC, 1; without IDC, 3), *C*. *dubliniesnsis* (without IDC, 1) and unclassified (with IDC, 2; without IDC, 5).

^d^Polymicrobial bacteremia/fungemia were included due to different species of *Candida* spp. (with IDC, 6; without IDC, 6) and due to pathogens other than *Candida* spp. (with IDC, 11; without IDC, 2)

The IDC group (55.6% [70/126]) had significantly more patients with candidemia during 2010–2013 than non-IDC group (21.0% [33/157]) (*P* < 0.001). Although there was a similar profile of chronic conditions in both groups, patients in the IDC group were significantly more associated with HIV infection (*P* = 0.006) and connective tissue disease (*P* = 0.018). There were no differences of healthcare-associated exposures between the two groups. The IDC group had more previous exposure to carbapenems (*P* = 0.014), metronidazole (*P* = 0.023), micafungin (*P* = 0.020), and liposomal amphotericin b (*P* = 0.038). Although there was no difference of isolated *Candida* spp. between groups, the IDC group had significantly more episodes of polymicrobial bacteraemia/fungaemia than the non-IDC group (*P* = 0.013). The proportion of PLABSI was significantly higher in the IDC group (*P* = 0.020). No differences of severe sepsis/septic shock and chorioretinitis were observed between groups, but patients in the IDC group developed significantly more acute renal failure than did non-IDC group (*P* = 0.034). Regarding treatment, the IDC group more frequently received micafungin (*P* < 0.001) as empiric therapy. In contrast, the number of patients who received fluconazole or who did not receive any empiric therapy was higher in the non-IDC group (*P* = 0.001 for both). Patients with IDC were more likely to receive the appropriate definitive antifungal therapy (*P* < 0.001) and appropriate duration of antifungal therapy (*P* < 0.001). Although there was no difference in the overall adequate source control between groups, early CVC removal and early peripheral-line removal were more frequent in patients with IDC than patients without (*P* = 0.023, and *P* = 0.013, respectively). Patients with IDC received more consultations from an ophthalmologist to evaluate chorioretinitis (*P* < 0.001) (Table 2).

**Table 2:** Parameters associated with recommended candidemia treatment and results, n = 283.

Variable		With IDC		Without IDC		OR (95% CI)		*P* value
		(n = 126, 44.5%)		(n = 157, 55.1%)				
Definitive antifungal therapy	Appropriate antifungal choice (n = 276) ^a^	120/124	(96.8)	125/152	(82.2)	6.48	(2.20–19.07)	<0.001
	Liposomal amphotericin b	4	(3.2)	3	(2.0)	1.66	(0.36–7.54)	0.70
	Fluconazole	51	(41.1)	19	(12.5)	4.89	(2.69–8.90)	<0.001
	Echinocandin	9	(7.3)	12	(7.9)	0.91	(0.37–2.24)	0.84
	Voriconazole	0	(0.0)	3	(2.0)			0.26
	Appropriate planned duration of antifungal therapy (n = 280)^b^	105/125	(84.0)	89/155	(57.4)	3.89	(2.19–6.92)	<0.001
	Median duration of antifungal therapy^b^	19	(15–31)	14	(6–20)			<0.001
Intervention	Transthoracic echocardiogram	22	(17.5)	1	(0.6)	33.00	(4.38–248.59)	<0.001
	Adequate source control	106	(84.1)	130	(82.8)	1.10	(0.59–2.07)	0.77
	CVC removal	86	(68.3)	120	(76.4)	0.66	(0.39–1.12)	0.12
	Early CVC removal (≤ 48 hours)	65	(51.6)	102	(65.0)	0.58	(0.36–0.93)	0.023
	Peripheral-line removal	17	(13.5)	8	(5.1)	2.91	(1.21–6.97)	0.013
	Intra-abdominal drainage	3	(2.4)	2	(1.3)	1.89	(0.31–11.49)	0.66
	Consultation to Ophthalmologist	98	(77.8)	54	(34.4)	6.68	(3.92–11.38)	<0.001
	Performed the ecocardiography	22	(17.5)	1	(0.6)	33.00	(4.38–248.59)	<0.001

Unless otherwise stated, data are presented as n (%)

IDC, infectious disease consultation; OR, odds ratio; CI, confidence interval; IQR, interquartile range; CVC, central venous catheter

^a^Appropriate antifungal choice was unspecified for 7patients due to unclassified *Candida* species.

^b^Duration of therapy was unspecified for 3 patients.

**Fig 1:**
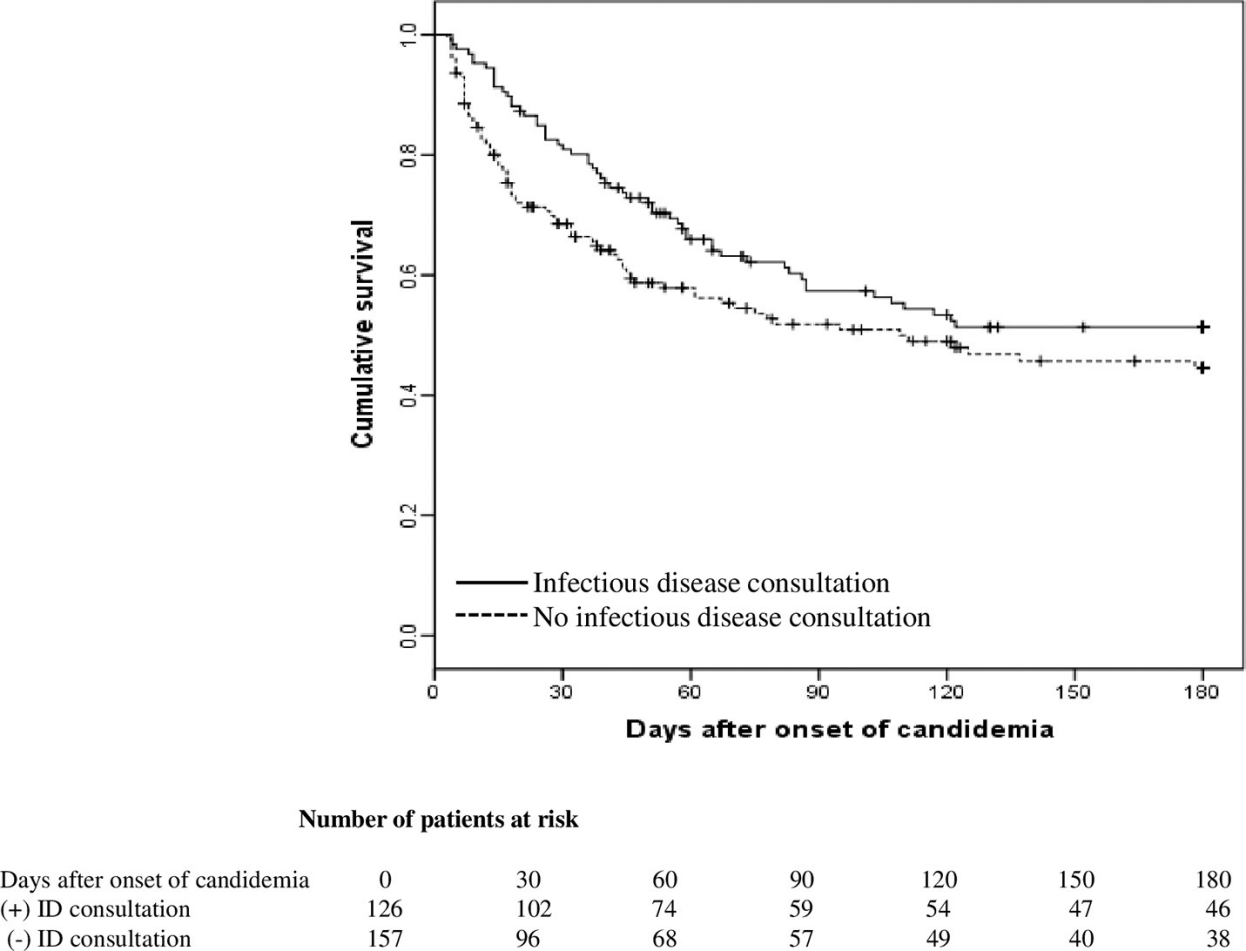
Kaplan-Meier curve of candidemia based on infectious disease consultation. Kaplan-Meier curve of candidemia based on infectious disease consultation. Adjusted hazard ratio for 30-day mortality was 0.54 (*P* = 0.017), and the crude hazard ratio for 31-180-day mortality was 0.98 (*P* = 0.92).

The overall 30-day and 180-day all-cause mortality were 23.7% (67/283) and 47.0% (133/283), respectively. The median length of time from diagnosis to death was 26 (IQR, 11–55) days. No difference in hospital mortality between two groups, but the IDC group had more episodes of persistent candidemia (*P* < 0.001) with longer total LOS (*P* < 0.001) and longer duration candidemia (*P* <0.001) (Table 1). The Kaplan-Meier survival curve for patients with candidemia stratified by IDC is shown in Fig 1.

### Predictive factors for mortality of candidemia

Predictive factors for mortality of candidemia are summarized in Table 3.

**Table 3:** Predictive factors for 30-day mortality of candidemia patients, n = 283.

	No. (%) of patients with:				Univariate analysis			Multivariate analysis		
	Died ≤ 30 day after the onset		Survived > 30 day after the onset		Crude HR		*P* value	Adjusted HR		*P* value
Variable	(n = 67, 23.7%)		(n = 216, 76.3%)		(95% CI)			(95% CI)		
Mean age (years) ± SD	73.6	± 12.0	68.9	± 16.7	1.02	(1.00–1.04)	0.035	1.01	(1.00–1.03)	0.15
Females	24	(35.8)	65	(30.1)	1.27	(0.77–2.10)	0.35			
Candidemia from 2010 to 2013	24	(35.8)	79	(36.6)	0.97	(0.55–1.60)	0.90			
HIV infection	1	(1.5)	9	(4.2)	0.37	(0.051–2.63)	0.32			
Neutropenia (< 0.5 × 10^9^ cells/L) at onset	3	(4.5)	4	(1.9)	2.03	(0.90–4.60)	0.090			
Chemotherapy in the past month	14	(20.9)	35	(16.2)	1.28	(0.71–2.30)	0.42			
Steroid therapy in the past month	15	(22.4)	36	(16.7)	1.28	(0.72–2.27)	0.40			
Radiation therapy in the past month	4	(6.0)	14	(6.5)	0.96	(0.35–2.64)	0.92			
Transplantation in the past month	0	(0.0)	4	(1.9)	0.049	(0.0–215.90)	0.48			
Dependent functional status	33	(49.3)	88	(40.7)	1.39	(0.86–2.24)	0.18			
Charlson's weighted index comorbity score (6), mean ± SD	4.3	± 2.8	4.2	± 2.9	1.00	(0.92–1.09)	0.97	1.01	(0.93–1.10)	0.76
Diabetes mellitus	20	(29.9)	56	(25.9)	1.14	(0.67–1.92)	0.63			
Solid-organ cancer within last 1 year	27	(40.3)	84	(38.9)	1.05	(0.65–1.72)	0.84			
Hematological malignancy within last 1 year	7	(10.4)	18	(8.3)	1.18	(0.54–2.58)	0.68			
Chronic kidney disease stage V	6	(9.0)	12	(5.6)	1.58	(0.69–3.67)	0.28			
Liver diseases	1	(1.5)	13	(6.0)	0.28	(0.039–2.00)	0.20			
Chronic heart disease	20	(29.9)	39	(18.1)	1.70	(1.01–2.87)	0.047			
Chronic obstructive pulmonary disease	11	(16.4)	22	(10.2)	1.57	(0.82–2.99)	0.17			
Cerebrovascular disease	17	(25.4)	46	(21.3)	1.22	(0.70–2.11)	0.48			
Dementia	3	(4.5)	17	(7.9)	0.62	(0.20–1.98)	0.42			
Connective tissue disease	4	(6.0)	12	(5.6)	0.99	(0.36–2.72)	0.98			
Peptic ulcer disease	6	(9.0)	20	(9.3)	0.93	(0.40–2.14)	0.86			
Peripheral vascular disease	2	(3.0)	2	(0.9)	2.98	(0.73–12.19)	0.13			
Hemiplegia	3	(4.5)	21	(9.7)	0.47	(0.15–1.48)	0.20			
Resided in LTCF in the past 3 months	1	(1.5)	13	(6.0)	0.28	(0.038–2.00)	0.20			
Hospitalized in the past 3 months	37	(55.2)	93	(43.1)	1.61	(0.99–2.60)	0.054			
Invasive procedure/surgery in the past 3 months	18	(26.9)	82	(38.0)	0.66	(0.38–1.13)	0.13			
Tracheotomy in the past 3 months	8	(11.9)	23	(10.6)	1.06	(0.51–2.21)	0.88			
Urinary catheters (for ≥ 2 days) at onset of candidemia	57	(85.1)	122	(56.5)	3.72	(1.90–7.27)	< 0.001	2.94	(1.48–5.87)	0.002
CVC (for ≥ 2 days) at same onset	59	(88.1)	172	(79.6)	1.76	(0.84–3.68)	0.14			
Median days of CVC prior to onset of candiedemia (IQR)	15	(9–27)	11	(4–23)	1.01	(1.00–1.01)	< 0.001			
Undergoing hemodialysis in the past month	6	(9.0)	16	(7.4)	1.16	(0.50–2.69)	0.72			
Transfusion in the past month	41	(61.2)	113	(52.6)	1.29	(0.79–2.10)	0.32			
TPN in the past month	43	(64.2)	129	(59.7)	1.21	(0.73–1.99)	0.47			
Median days of TPN prior to onset of candidemia (IQR)	10	(0–20)	6	(0–16)	1.01	(1.00–1.02)	0.26			
ICU stay in current hospitalization before onset of candidemia	10	(14.9)	26	(12.0)	1.16	(0.59–2.27)	0.67			
Median hospital days prior to the onset of candidemia (IQR)	38	(20–68)	34	(18–66)	1.00	(0.99–1.00)	0.53			
Over all	63	(94.0)	204	(94.4)	0.97	(0.35–2.67)	0.96			
Penicillins^a^	27	(40.3)	95	(44.0	0.89	(0.55–1.46)	0.65			
Cephalosporins^b^	27	(40.3	103	(47.7	0.79	(0.49–1.29)	0.35			
Carbapenems	35	(52.2)	106	(49.1)	1.08	(0.67–1.75)	0.74			
Fluoroquinolones	18	(26.9)	49	(22.7)	1.18	(0.69–2.03)	0.54			
Aminoglycosides	8	(11.9)	20	(9.3)	1.23	(0.59–2.58)	0.58			
Trimethoprim-sulfamethoxazole	5	(7.5)	28	(13.0)	0.55	(0.22–1.37)	0.20			
Clindamycin	9	(13.4)	22	(10.2)	1.25	(0.62–2.52)	0.54			
Metronidazole	9	(13.4)	15	(6.9)	1.86	(0.92–3.75)	0.084			
Glycopeptide	28	(41.8)	59	(27.3)	1.65	(1.01–2.68)	0.044			
Over all	11	(16.4)	27	(12.5)	1.27	(0.66–2.42)	0.48			
Fluconazole	0	(0.0)	12	(5.6)	0.046	(0.0–6.19)	0.22			
Micafungin	10	(14.9)	12	(5.6)	2.32	(1.18–4.54)	0.014			
Voriconazole	0	(0.0)	2	(0.9)	0.049	(0.0–6882.16)	0.62			
Liposomal amphotericin b	0	(0.0)	4	(1.9)	0.049	(0.0–215.90)	0.48			
Itraconazole	2	(3.0)	6	(2.8)	1.09	(0.27–4.43)	0.91			
*C*. *albicans*	37	(55.2)	94	(43.5)	1.51	(0.93–2.45)	0.093			
*C*. *glabrata*	16	(23.9)	58	(26.9)	0.89	(0.51–1.56)	0.68			
*C*. *parapsilosis*	5	(7.5)	40	(18.5)	0.39	(0.16–0.96)	0.040	0.57	(0.22–1.45)	0.24
*C*. *tropicalis*	10	(14.9)	18	(8.3)	1.69	(0.86–3.30)	0.13			
Others^c^	3	(4.5)	14	(6.5)	0.69	(0.22–2.20)	0.53			
Polymicrobial bacteremia/fungemia^d^	11	(16.4)	14	(6.5)	2.07	(1.08–3.95)	0.028	0.55	(0.13–2.31)	0.42
Previous *Candida* colonization within one week before candidemia	23	(34.3)	67	(31.0)	1.13	(0.68–1.86)	0.65			
CLABSI	58	(86.6)	162	(75.0)	2.02	(1.00–4.07)	0.050	1.47	(0.62–3.47)	0.38
PLABSI	3	(4.5)	32	(14.8)	0.30	(0.094–0.95)	0.041	0.55	(0.13–2.31)	0.42
Intra-abdominal infection	2	(3.0)	11	(5.1)	0.57	(0.14–2.31)	0.43			
Unknown source	4	(6.0)	11	(5.1)	1.18	(0.43–3.23)	0.75			
Sepsis	15	(22.4)	77	(35.6)	0.57	(0.32–1.01)	0.053			
Severe sepsis	33	(49.3)	75	(34.7)	1.68	(1.04–2.71)	0.034			
Septic shock	16	(24.2)	23	(10.6)	2.16	(1.23–3.79)	0.007			
Sever sepsis/septic shock	49	(73.1)	98	(45.4)	2.78	(1.62–4.77)	< 0.001	2.10	(1.20–3.65)	0.009
Reduced consciousness	17	(25.4)	16	(7.4)	3.09	(1.78–5.37)	< 0.001			
Acute mechanical intubation/ventilation	14	(20.9)	17	(7.9)	2.29	(1.27–4.13)	0.006			
Developed acute renal failure	23	(34.3)	33	(15.3)	2.38	(1.44–3.94)	0.001			
Developed acute liver injury	27	(40.3)	68	(31.5)	1.36	(0.83–2.21)	0.22			
Chorioretinitis	4	(6.0)	29	(13.4)	0.42	(0.15–1.16)	0.095			
Consultation to ID specialist	23	(34.3)	103	(47.7)	0.56	(0.34–0.93)	0.025	0.54	(0.32–0.90)	0.017

Unless otherwise stated, data are presented as n (%)

HR, hazard ratio; CI, confidence interval; SD, standard deviation; LTCF, long term care facility; CVC, central venous catheter; IQR, interquartile range; TPN total parenteral nutrition; CLABSI, central line-associated blood stream infection; PLABSI, peripheral line-associated blood stream infection; ID, infectious disease

^a^Included ampicilline, sulbactam/ampicilline, piperacillin, and tazobactam/piperacillin

^b^Included ceftriaxone, ceftazidime, and cefepime.

^c^Other *Candida* species were included *C*. *gelliermondii* (survived, 3), *C*. *lusitaniae* (survived, 3), *C*. *krusei* (survived, 3; died, 1), *C*. *dubliniesnsis* (died, 1) and unclassified (survived, 6; died, 1).

^d^Polymicrobial bacteremia/fungemia were included due to different species of *Candida* spp. (survived, 8; died, 7) and due to pathogens other than *Candida* spp. (survived, 6; died, 4)

Patients with candidemia who died within 30 days of the first positive blood culture were more likely to be older (*P* = 0.035), to have chronic heart disease (*P* = 0.047), to have urinary catheters placed (*P* < 0.001), to have exposure to glycopeptide (*P* = 0.044), to have polymicrobial bacteremia/fungemia (*P* = 0.028), to have CLABSI (*P* = 0.050), and to be in severe sepsis/septic shock (*P* < 0.001). They were less likely to have candidemia due to *C*. *parapsilosis* (*P* = 0.040) and to have PLABSI (*P* = 0.041) than were candidemia patients who survived longer than 30 days after candidemia onset. In the multivariate model, factors independently associated with 30-day mortality among candidemia were urinary catheters use at onset (adjusted hazard ratio [HR] = 2.94; 95% CI = 1.48–5.87; *P* = 0.002) and severe sepsis/septic shock (adjusted HR = 2.10; 95% CI = 1.20–3.65; *P* = 0.009). IDC was associated with a decreased risk of 30-day mortality (adjusted HR = 0.54; 95% CI = 0.32–0.90; *P* = 0.017).

For the 216 patients who survived 30 days after initial positive culture, the effect of IDC from 31- to 180-day of the diagnosis of candidemia was also evaluated. There was no statistical difference in 31-180-day mortality after the diagnosis of candidemia between patients who did and did not receive IDC (crude HR = 0.98; 95% CI = 0.60–1.58; *P* = 0.92).

## Discussion

We showed that IDC was associated with a 46% reduction in all-cause mortality among candidemia patients within 30 days of candidemia onset. Past studies have described the positive effect of IDC on mortality with candidemia; 18–24% and 39–56% in the IDC and the non-IDC groups, respectively [13–16]. Compared the data of rates of IDC, patient characteristics at baseline, and clinical outcomes of candidemia in a hospital in North America [16], the rate of IDC was lower (45% vs 77%), the median age and population of male were higher (69 years old vs 53 years old; 72% vs 55%), and 30 days mortality was slightly lower (18% vs 20%) among IDC group in our results. However, these studies were limited due to small sample sizes (50–171 patients) and short study periods (1–3 years). Although our study was a retrospective cohort study, our sample size was larger (283 patients) and study period was longer (12 years) than past studies [13–16]. Moreover, because previous studies did not use the cox proportional hazard model [13–16], the evaluation of the effect of IDC on mortality with candidemia was prone to confounding effects, and might thus be incorrect. In our study, the IDC group more frequently received appropriate choice (OR = 6.48; 95% CI = 2.20–19.07; *P* < 0.001) and appropriate duration (OR = 3.89; 95% CI = 2.19–6.92; *P* < 0.001) of antifungal therapy than the non-IDC group. The duration of antifungal therapy was significantly longer in the IDC group than the non-IDC group (19 days versus 14 days; *P* < 0.001). Past research has also revealed that IDC intervention for candidemia led to appropriate antifungal therapy [14–16].

Besides antifungal therapy, adequate source control is an important component of appropriate candidemia management. While previous research has reported that removal of CVC was more frequent in the IDC group than in the non-IDC group [13–16, 26], our results showed no statistical difference in numbers of removal of CVC between both groups. In our study, we found that the IDC group had a statistically higher proportion of the removal of peripheral-line than the non-IDC group (OR = 2.91; 95% CI = 1.21–6.97; *P* = 0.013), which, to the best of our knowledge, has not been reported previously. This might reflect the difficulty of diagnosing PLAC, and need for the consultation of an ID specialist [17]. In fact, the proportion of PLAC was higher in the IDC group than the non-IDC group (OR = 2.34; 95% CI = 1.13–4.87; *P* = 0.020). Similar to a past study [26], our study revealed that consultation with an ophthalmologist was conducted more often in the IDC group then in the non-IDC group (OR = 6.68; 95% CI = 3.92–11.38; *P* < 0.001). For candidemia, the evaluation of endophthalmitis is thought to be a predictive factor for outcome improvement of candidemia, and contributes to an appropriate choice and duration of antifungal therapy [20, 27]. While not statistically significant, the proportion of endophthalmitis was higher in the IDC group than the non-IDC group in our study (OR = 1.58; 95% CI = 0.76–3.27; *P* = 0.22), suggesting that IDC led to the detection of endophthalmitis in many cases.

On the other hand, urinary catheters placed (for ≥ 2 days) at onset of candidemia (adjusted HR, 2.94; 95% CI, 1.48–5.87, *P* = 0.002) and severe sepsis/septic shock (adjusted HR, 2.10; 95% CI, 1.20–3.65, *P* = 0.009) were independently associated with increased 30-day mortality. These results indicate that IDC might not significantly improve outcomes for patients with very severe conditions. Clinical severity such as severe sepsis/septic due to candidemia was previously reported as an independent risk factor for 30-day mortality [28].

The IDC group was associated with the following factors indicating the clinical severity of candidemia: ICU stay, development of acute renal failure, exposure to carbapenems/micafungin/liposomal amphotericin b, longer duration of candidemia, and longer length of hospital stay after candidemia [28]. In the IDC group, episodes of polymicrobial bacteremia/fungemia were statistically more frequently found than in the non-IDC group (OR = 2.91; 95% CI = 1.21–6.97; *P* = 0.013). This may be due to heavier contamination of peripheral lines than central lines, and better ability of ID specialists for diagnosing PLAC [17]. IDC as candidemia intervention may not improve the severity of the current disease status, however it did improve the mortality of candidemia, with improved management of the disease.

This study has several limitations. Due to the retrospective nature of the study, we were unable to collect information regarding the duration of time from onset of candidemia to IDC. This is a time-dependent variable, and might have been an unmeasured confounding factor. Similarly, unmeasured confounding factors such as other interventions might have affected the patients’ outcomes. Following the recommendation of IDC for candidemia was not mandatory. Although IDC consisted of chart review, physical examination of the patient, follow-up visits, and written recommendations for therapy based on published IDSA guidelines, final decision-making in each case depended on the primary team in charge.

In conclusion, this study was the first epidemiological clinical study with a large sample size and a long study period to evaluate the value of IDC in candidemia in Japan. IDC was associated with a 46% reduction in adjusted all-cause mortality among candidemia patients within 30 days of onset of candidemia. These results suggest that IDC should be actively considered to improve the frequently poor outcome of candidemia patients. Further studies, including evaluations of the outcome effects of time between IDC and onset of candidemia, are needed to further reduce the mortality of candidemia.

## Supporting information

S1 Table A data sheet of the present study. (XLSX)
